# Appropriateness of Diagnostic Coronary Angiography as a Measure of Cardiac Ischemia Testing in Non-Emergency Patients – A Retrospective Cross-Sectional Analysis

**DOI:** 10.1371/journal.pone.0117172

**Published:** 2015-02-26

**Authors:** Corinne Chmiel, Oliver Reich, Andri Signorell, Ryan Tandjung, Thomas Rosemann, Oliver Senn

**Affiliations:** 1 Institute of Primary Care, University of Zurich, Zurich, Switzerland; 2 Department of Health Sciences, Helsana Group, Zurich, Switzerland; University of Messina, ITALY

## Abstract

**Background:**

Adequate application of guidelines concerning non-invasive ischemia testing (NIIT) could avoid inappropriate invasive testing in non-emergency situations. Hardly any data exists regarding frequency and appropriateness of diagnostic coronary angiography (CA). The aim of this study was to evaluate the proportion and predictors of patients without NIIT prior to elective purely diagnostic CA without therapeutic intervention.

**Methods:**

Retrospective cross-sectional analysis of insurance claims data from 2012 and 2013. Patients <18 years, acute cardiac ischemia and emergency procedures and patients insured in a managed care model were excluded from analysis. The proportion of patients with NIIT procedures (stress-ECG, transthoracic echocardiography, stress echocardiography, scintigraphy, computer tomography, heart MRI) undertaken within two months before diagnostic CA was assessed. Multiple logistic regression analysis was applied to investigate independent determinants for receiving NIIT.

**Findings:**

2714 patients were included for analysis. 37.5% (1018) did not receive any NIIT before CA. When high risk patients (patients having received therapeutic cardiac intervention within one month after or 18 months prior to diagnostic CA, n = 766) were excluded 34.3% (669) did not receive NIIT before CA. High risk status as well as >6 chronic comorbidities were independently associated with a lower proportion of NIIT (p<0.0001, OR 0.607 and p = 0.0041, OR 0.648), when additionally controlled for age, sex, language area, insurance coverage, inpatient treatment, cardiovascular medication and lower number of chronic comorbidities. Age (p<0.05, OR 1.009) and intake of oral antiplatelet therapy (p<0.0001, OR 1.914) were independently associated with a higher proportion of NIIT when controlled for the mentioned cofactors.

**Conclusions:**

Our data show that despite the existence of guidelines a substantial overuse of a potentially harmful and inappropriate diagnostic intervention is performed suggesting the need for improvement of diagnostic pathways prior to invasive testing.

## Introduction

Recommendations vary regarding the optimal approach to patients with suspected coronary heart disease (CHD) in a non-emergency setting. The American Heart Association suggests non-invasive ischemia testing (NIIT) for all patients with suspected CHD or change in clinical status in a patient with known CHD unless unstable angina pectoris is assumed [1]. The British (NICE) [2,3] guidelines recommend using imaging studies based on CHD risk scoring and not stress tolerance test to exclude angina in patients with no previous history of CHD. For patients with an estimated likelihood for CHD of 61–90% in whom stable angina cannot be diagnosed or excluded by clinical assessment alone, invasive coronary angiography (CA) after performing a 12-lead ECG is suggested. The European Society of Cardiology recommends no testing by means of non-invasive methods in patients with a pre-test probability below 15% or above 85%. In such patients, either no obstructive or obstructive coronary artery can be assumed [4]. The Swiss Society of Cardiology does not publish any recommendations concerning diagnostic pathway prior to CA except in the case of acute coronary syndrome. Adequate application of guidelines concerning non-invasive ischemia testing could avoid inappropriate invasive testing. Nevertheless a few studies from the U.S.A indicate that a large proportion of patients does not undergo recommended NIIT prior to CA, raising the question whether a substantial amount of patients might currently undergo inappropriate invasive testing with associated costs [5–8].

CA has long been considered to be the best test for the detection of CHD. Significant CHD is postulated in case of luminal narrowing of a coronary artery of at least 50%. Concerns over clinical usefulness of CA have been raised, since an atherosclerotic coronary artery lesion need not be obstructive to become thrombogenic, nor do all obstructive lesions have thrombogenic potential. Angiographic findings do not necessarily accurately predict the site of lesions that leads to subsequent coronary artery thrombosis. Therefore coronary bypass surgery or angioplasty directed only at discernible stenotic lesions may not be effective for preventing subsequent myocardial infarctions [9]. Not only the uncertainty about clinical relevance of the findings in CA, but also the associated risks of this intervention should be taken into consideration, before invasive diagnostic testing is performed. Major complication rates have been estimated between 0.1% and 3%, including procedure-related mortality, non-fatal Q wave myocardial infarction, coronary artery spasm, severe arrhythmia, severe contrast allergic reaction, retroperitoneal haemorrhage, peripheral vascular complication, acute kidney injury (contrast nephropathy) and heart failure [10]. Besides these major risks also minor but common complications at the site of catheter insertion should be considered including acute thrombosis, distal embolization, vascular dissection, poorly controlled bleeding, hematoma, pseudoaneurysm or arteriovenous fistula [11–13]. Compared to CA, NIIT on the other hand has the capability of providing prognostic information by estimating the ischemic effect of a coronary lesion and has been shown to reduce the use of CA as well as associated costs [8,14]. Utilisation of NIIT in combination with selective CA therefore results in optimal yield of diagnostic and therapeutic CA.

No data exist on frequency and appropriateness of diagnostic CA in Europe. The aim of this study was therefore to evaluate the proportion and predictors of patients without NIIT prior to elective purely diagnostic CA without therapeutic intervention. We performed a retrospective analysis of insurance claims data on diagnostic procedures undertaken within two months before CA.

## Methods

### Setting

In Switzerland, patients have mandatory health insurance, which provides unlimited access to the health care system, including specialist care and emergency care at the hospital. Unlike other European countries, there are no mandatory barriers (gate-keeping systems). Depending on the insurance model chosen by the patient, annual deductibles for adults vary between 300 and 2500 Swiss Francs and access to specialists might be limited. In limited access models, such as the managed care model for example, the general practitioner or the insurance telephone hotline has to be consulted before contacting a specialist or another institution such as a hospital. In case of emergency this regulation is overruled.

### Subjects, data collection and measurements

Data for this study included mandatory health insurance claims from approximately 1.2 million persons which lived all over Switzerland and were enrolled with the Helsana Group, consisting of four health insurance plans. Data on patients undergoing CA in the years 2012 and 2013 were retrospectively analysed.

#### Inclusion criteria

Purely diagnostic uncomplicated CA in adults in the years 2012 and 2013 (all patients enrolled were not treated with coronary angioplasty/stenting or coronary artery by-pass grafting):

- In-patients with SwissDRG-Code F49F (invasive cardiologic diagnostics except in acute myocardial infarction, one hospital day stay).
- Outpatients: Tarmed positions (Standard billing rate for outpatient medical care in Switzerland): 17.0710 (CA, basic service I) and/or 17.0740 (CA, arterial approach, basic service II) and/or 17.1010 (left heart catheterization) and/or 17.1090 (selective CA) and/or 17.1810 (technical basic service 0, CA/cardiologic- interventional radiology) and/or 17.1820 (technical basic service 1, CA/cardiologic- interventional radiology).

#### Exclusion criteria

Emergency CA. CA was defined as emergency procedure when an emergency Tarmed position was charged for at the same day as the procedure was performed (positions 00.2510, 00.2520, 00.2540, 00.2560, 00.2580, 35.0610). In 198 cases this criteria was met.

- To exclude selection bias in a health care system with no mandatory gate-keeping system, which is the case in Switzerland, patients insured voluntarily in a managed care model were excluded from analysis. In 2531 cases this criteria was met.

#### Measurements

Patient characteristics: sex, age, language area and type of insurance coverage (deductible class, supplementary private hospital insurance)

- Setting of CA: inpatient or outpatient
- Diagnostic procedures performed within two months prior to CA according to Tarmed position:
- 17.0010: Electrocardiogram (ECG): not considered as NIIT, only in combination with other NIIT
- 17.0050: Cardiac intervention with medication under continuous registration of ECG: not considered as NIIT, only in combination with another NIIT
- 17.0060: ECG performed by specialist outside of the practice or hospital: not considered as NIIT, only in combination with another NIIT
- 17.0080 and 17.0090: Stress-ECG
- 17.0210: Echocardiography, transthoracic, qualitative and quantitative examination of adult
- 17.0280: Stressechocardiography, physical stress
- 17.0290: Stressechocardiography, medication stress
- 31.0260: Scintigraphy physiologicily triggered
- 39.4060: Computed tomography of entire thorax and/or sternoclavicular joint
- 39.5100 Heart MRI
- Cardiovascular Medication grouped according to Anatomical-Therapeutic-Chemical-Classification (ATC) [15]
- Group 1: N02BA01, B01AC (Aspirin, platelet aggregation inhibitors)
- Group 2: C10* (statins, lipid modifying agents)
- Group 3: C02*, C03*, C07*, C08*, C09* (antihypertensives, diuretics, beta blocking agents, calcium channel blockers, agents acting on the renin-angiotensin system)
- Group 4: A10* (antidiabetics)
- Number of chronic conditions according to Pharmaceutical cost groups PCG [16]
- Group 1: 3–4 chronic diseases
- Group 2: 5–6 chronic diseases
- Group 3: more than 6 chronic diseases

#### Sensitivity Analysis with high risk patients

We performed a sensitivity analysis of our data by defining a subgroup of patients as high risk patients with supposed cardiac disease (total n = 766) if having received following therapeutic cardiac intervention/diagnosis within one month after and/or 18 months prior to diagnostic CA:

- Up to 18 months prior to CA registered SwissDRG-Code from chapter F (diseases and dysfunction of the circulatory system) or Tarmed position 17.1110 (percutaneous transluminal coronary angioplasty in coronary stenosis or coronary occlusion, first dilated vessel segment) or 17.1240 (angioplasty of cardiac arteries and veins, first dilated vessel) (n = 473)
- Up to one month after CA registered SwissDRG-Code from the chapter F (diseases and dysfunction of the circulatory system) or Tarmed positions 17.1110 (percutaneous transluminal coronary angioplasty in coronary stenosis or coronary occlusion, first dilated vessel segment) or 17.1240 or (angioplasty of cardiac arteries and veins, first dilated vessel) (n = 242)
- Combination of the two options (n = 51)

### Processing and analysing data

Data was checked for eligibility and completeness and subjected to a set of predefined plausibility tests. These included checks for contradictory data, duplication and plausibility of time measurements.

### Statistical analysis

Descriptive statistical techniques were used to provide a general profile of the study population and grouped into patients with or without NIIT. These data were presented as means in the case of continuous variables and as percentages in case of categorical variables. Furthermore, differences between the two groups with respect to prior NIIT in terms of demographics, insurance coverage, number of chronic conditions, medication class and language area were analysed with a nonparametric analysis of variance (Kruskal-Wallis test for continuous variables and chi-square tests for categorical variables). We developed several statistical models to evaluate the major outcome of receiving NIIT within two months prior CA. In order to assess patient-level effects the following independent variable were included in the models: age, sex, deductible class, supplementary private hospital insurance coverage, language area, inpatient CA, cardiac medication class according to ATC, number of chronic medical conditions identified using PCGs and high risk status for CHD. The strength of associations was measured by the odds ratio (OR) and the respective 95% confidence intervals (CI). The level of significance was set at 0.05. All statistical analyses were performed using R version 3.1.0 (2014–04–10) (R Foundation for Statistical Computing, Vienna, Austria).

### Ethics approval

According to the national ethical and legal regulation, an ethical approval was not needed. Permission to access the study data was provided by the Helsana Group. Since data was anonymized, no consent of patients was required.

## Results

6269 (diagnostic and therapeutic) non-emergency CA were performed in 2012 and 2013. Overall a total of 2714 patients met the predefined inclusion and exclusion criteria and were therefore included for analysis.766 of these were considered high risk patients (Table 1).

**Table 1:** Descriptive statistics of study population.

	**Total Population**				**High Risk ° Patients excluded**				
	**Total**	**No NIIT**	**With NIIT**		**Total**	**No NIIT**	**With NIIT**	
Count	2714	1018 (37.5%)	1696 (62.5%)		1948	669 (34.3%)	1279(65.7%)	
Age (Years)	66.1	65.4	66.6		65.3	63.7	66.2	*
Sex (Female)	1026 (37.8%)	374 (36.7%)	652 (38.4%)		762 (39.1%)	243 (36.3%)	519 (40.6%)	
Deductible Class (Swiss Francs)								
300	1818 (67.0%)	678 (66.6%)	1140 (67.2%)		1303 (66.9%)	447 (66.8%)	856 (66.9%)	
500	640 (23.6%)	244 (24.0%)	396 (23.3%)		462 (23.7%)	159 (23.8%)	303 (23.7%)	
1000	52 (1.9%)	24 (2.4%)	28 (1.7%)		40 (2.1%)	16 (2.4%)	24 (1.9%)	
1500	99 (3.6%)	32 (3.1%)	67 (4.0%)		73 (3.7%)	22 (3.3%)	51 (4.0%)	
2000	7 (0.3%)	3 (0.3%)	4 (0.2%)		6 (0.3%)	3 (0.4%)	3 (0.2%)	
2500	98 (3.6%)	37 (3.6%)	61 (3.6%)		64 (3.3%)	22 (3.3%)	42 (3.3%)	
Supplementary private insurance	711 (26.2%)	255 (25.0%)	456 (26.9%)		528 (27.1%)	178 (26.6%)	350 (27.4%)	
French or Italian part of Switzerland	880 (32.4%)	323 (31.7%)	557 (32.8%)		625 (32.1%)	198 (29.6%)	427 (33.4%)	
Inpatient CA	1278 (47.1%)	479 (47.1%)	799 (47.1%)		880 (45.2%)	279 (41.7%)	601 (47.0%)	*
ATC_Group 1	1219 (44.9%)	366 (36.0%)	853 (50.3%)	*	859 (44.1%)	220 (32.9%)	639 (50.0%)	*
ATC_ Group 2	947 (34.9%)	349 (34.3%)	598 (35.3%)		643 (33.0%)	217 (32.4%)	426 (33.3%)	
ATC_ Group 3	1702 (62.7%)	634 (62.3%)	1068 (63.0%)		1185 (60.8%)	396 (59.2%)	789 (61.7%)	
ATC_ Group 4	403 (14.8%)	152 (14.9%)	251 (14.8%)		280 (14.4%)	91 (13.6%)	189 (14.8%)	
Number of chronic conditions (PCG)	4.5	4.6	4.4	*	4.4	4.6	4.3	*

Anatomical-Therapeutic-Chemical-Classification (ATC) group 1 = Aspirin, platelet aggregation inhibitors, Group 2 = statins, lipid modifying agents, group 3 = antihypertensives, diuretics, beta blocking agents, calcium channel blockers, agents acting on the renin-angiotensin system, group 4 = antidiabetics. Coronary Angiography (CA). Non-invasive ischemia testing (NIIT). Pharmaceutical cost groups (PCG).

°High risk patients: having received therapeutic cardiac intervention within one month after or 18 Months prior to diagnostic CA.

*p<0.05.

### Non-invasive ischemia testing

37.5% (1018) did not receive NIIT before CA. When excluding high risk status 34.3% (669) did not receive NIIT (Table 1). 30.1% of patients without NIIT had a conventional ECG prior to CA, in the high risk population this was the case in 29% The most common NIIT combination in both populations was stress-ECG + transthoracic echocardiography (22.0 and 24.0%) (Table 2).

**Table 2:** Most common non-invasive ischemia testing performed prior to coronary angiography.

	**Total population**					**High risk ° patients excluded**				
	**Total**	**No NIIT**	%	**With NIIT**	%	**Total**	**No NIIT**	%	**With NIIT**	%
Count	2714	1018	37.5	1696	62.5	1948	669	34.3	1279	65.7
Stress-ECG + Transthoracic Echocardiography				598	22.0				468	24.0
Transthoracic Echocardiography				405	14.9				282	14.5
Stress-ECG				307	11.3				244	12.5
Computer Tomography				56	2.1				40	2.1
Stress-ECG + Echocardiography + Computer Tomography				50	1.8				40	2.1

°High risk patients: having received therapeutic cardiac intervention within one month after or 18 Months prior to diagnostic CA. Electrocardiogram (ECG). Non-invasive ischemia testing (NIIT)

### Determinants for non-invasive ischemia testing

Determining factors for receiving NIIT were age (p = 0.007, OR 1.011), female sex (p<0.05, OR 1.255) and intake of oral antiplatelet therapy (p<0.001, OR 2.102). Determining factor for not receiving NIIT was >6 chronic comorbidities (p = 0.0018, OR 0.569). The other factors (deductible classes, supplementary private insurance, language area, inpatient CA, ATC Groups 2, 3 and 4, up to 6 chronic conditions) were no significant determining factors for NIIT. When additionally controlling for high risk cardiac status, all the findings except female sex remained significant. High risk patients were significantly less likely to receive NIIT (p<0.0001, OR 0.607) (Table 3).

**Table 3:** Determinants for receiving non-invasive ischemia testing before coronary angiography, controlled for high risk patients.

	**OR**	**LCI**	**UCI**	**Significance**	
Age (Years)	1.009	1.002	1.016	0.0100	*
Sex (Female)	1.119	0.945	1.325	0.1907	
Deductible Class Swiss Francs (Reference 300)					
500	0.941	0.774	1.144	0.5399	
1000	0.634	0.357	1.126	0.1198	
1500	1.179	0.752	1.847	0.4725	
2000	0.596	0.128	2.776	0.5093	
2500	0.970	0.623	1.511	0.8929	
Supplementary private hospital insurance	1.046	0.864	1.265	0.6454	
French or Italian part of Switzerland	1.010	0.844	1.209	0.9106	
Inpatient CA	0.950	0.800	1.127	0.5563	
ATC_Group					
1	1.914	1.610	2.275	0.0000	*
2	0.945	0.787	1.135	0.5458	
3	0.944	0.791	1.126	0.5199	
4	1.061	0.838	1.343	0.6206	
Number of chronic conditions according to PCG (Reference 0–2)					
3–4	0.968	0.757	1.237	0.7937	
5–6	0.826	0.641	1.064	0.1384	
>6	0.648	0.482	0.872	0.0041	*
High Risk cardiac status°	0.607	0.509	0.723	0.0001	*

Odds ratio (OR). Lower Confidence Interval (LCI). Upper Confidence Interval (UCI). Coronary Angiography (CA). Anatomical-Therapeutic-Chemical-Classification (ATC) group 1 = Aspirin, platelet aggregation inhibitors, Group 2 = statins, lipid modifying agents, group 3 = antihypertensives, diuretics, beta blocking agents, calcium channel blockers, agents acting on the renin-angiotensin system, group 4 = antidiabetics. Pharmaceutical cost groups (PCG).

°High risk patients: having received therapeutic cardiac intervention within one month after or 18 months prior to diagnostic CA.

*p<0.05.

## Discussion

In our study population of elective CA with no therapeutic consequence (no coronary angioplasty/stenting or coronary artery by-pass grafting) more than one third (37.5%) of patients did not receive NIIT before diagnostic CA. When excluding high risk patients from this population 34.3% did not receive NIIT. High risk status as well as >6 chronic comorbidities were independently associated with a lower proportion of NIIT, age and intake of oral antiplatelet therapy with a higher proportion of NIIT.

### Appropriateness of diagnostic coronary angiography

In our study emergency CA were excluded from analysis. Our study population therefore presents a selection of patients receiving elective diagnostic CA with at least stable CHD or no CHD at all. Nevertheless an astonishing 37.5% of our patients with purely diagnostic CA not resulting in any invasive cardiac intervention did not receive any NIIT before CA. We also performed a sensitivity analysis in which we excluded high risk cardiac patients from analysis. Even in this subgroup the proportion of patients not receiving any NIIT remained high (34.3%). To the best of our knowledge this is the first study in Europe on appropriateness of CA. Most studies concerning the matter were performed in North America. Those data show varying rates of NIIT prior to CA. An insurance data based US study among Medicare patients showed a rate of 44.5% undergoing stress testing within 90 days prior to elective CA. Regional variation was large ranging from 22.1 to 70.6% [5]. Another US study among commercially insured populations showed a similar NIIT rate (only exercise stress test) to our study population (34.4% and 29%, respectively) [7]. These large amounts of diagnostic CA without prior NIIT found in literature as well as in our study population amaze in light of previous studies showing that stress testing prior to CA and angioplasty has been associated with lower overall diagnostic costs, shorter hospital stays and lower rates of revascularization, without adverse effects on cardiac death or myocardial infarction [7,8]. It has to be assumed that the inappropriate amount of CA in our study population could have most likely been avoided by performing NIIT prior to invasive procedure.

### Determinants for non-invasive ischemia testing

In our study female sex was initially associated with receiving NIIT prior to CA, possibly explainable by a lower pre-test probability of CHD in women. This finding was not sustainable when controlled for high risk cardiac status. Contrary to our study female sex was associated with a decreased likelihood of prior stress testing in the two US studies [5,7]. In those studies also older age was associated with a decreased likelihood of prior stress testing, whereas in our study on the contrary age was associated with a higher proportion of NIIT. Higher age represents a cardiac risk factor in the European and NICE guidelines, it would have therefore seemed reasonable that clinicians estimated older patients being at higher risk and therefore not performing NIIT prior to CA, according to the NICE and European guidelines. On the other hand the US guidelines suggest always performing NIIT independent of pretest-probability, therefore the finding of the US studies are also not consistent with their own regional guidelines. Clinicians could have also argued being more restrictive with invasive methods in older age, therefore rather performing NIIT. Since no other clinical data are available in our study, the full rationale for performing NIIT or not can only be speculated upon. The intake of oral antiplatelet therapy was also a robust determinant for receiving NIIT. This finding is difficult to interpret, since on the one hand in our study no clinical data exists, which would help estimate the conformity to European and NICE guidelines such as chest pain characteristics. On the other hand patients receiving an antiplatelet therapy are assumed to show a different cardiac risk profile than patients not receiving antiplatelet therapy, such as peripheral artery or cerebrovascular disease. Therefore physicians could have argued for immediate CA without NIIT when following the NICE and European Guidelines suggesting no NIIT for patients with a high cardiac risk profile. The presence of>6 comorbidities according to PCG was a robust determinant for not receiving NIIT, other than a lower amount of chronic comorbidities. This finding is similarly difficult to interpret as age, since we cannot estimate pretest probability and therefore the rationale of the clinicians. In the two US studies [5,7] as well as in our study high risk patients were robustly associated with decreased likelihood of NIIT. This seems to be a determinant which was clearly clinically reasonable and indicates that risk stratification was performed, considering the higher likelihood of a coronary pathology in patients with known CHD.

When analysing the pattern of determinants the conclusion arises that factors must have been involved in decision making which cannot be perceived by our study data and which probably are not part of guideline recommendations. Decision making according to perceived risk based on patient characteristics seems tricky not only in patients with stable CHD or non-cardiac chest pain. Lee et al have demonstrated in patients with non–ST-segment elevation myocardial infarction that referral for CA was based on the perceived risk, rather than objective evidence of ischemia [17]. Also in patients with stable angina evidence exists for the overestimation of CHD when current algorithms for cardiac risk stratification are applied: In the study of Cheng et al among patients which were referred for coronary computed tomographic angiography, determination of pretest likelihood of angiographically significant CHD by the invasive angiography-based guideline probabilities greatly overestimated the actual prevalence of disease [18].

### Guideline recommendations on diagnostic pathway

No specific type of NIIT is ideal. Depending on comorbidities the benefits of one NIIT outweigh the other. In the case of stable CHD only few randomized trials assessing health outcomes for diagnostic tests exist, therefore the available evidence has been ranked according to evidence from non-randomized studies or meta-analyses of these studies [4]. Even though the recently updated ESC/EACTS guidelines on the management of stable angina pectoris have downgraded the importance of stress ECG [4], a prudent approach in individuals with a suspicion of CHD who can exercise and who do not have ECG abnormalities seems still to be stress ECG testing as initial screening, when also taking easy accessibility and cost considerations into account. Nevertheless the different existing guidelines have controversial opinions on this matter depending on pre- and post-test probability. An exercise ECG also provides information on exercise capacity, which may be more predictive of outcome than ST segment changes [19,20]. International recommendations vary regarding the optimal approach to patients with suspected CHD [1–4]. Some always suggest NIIT prior to CA, except in emergency situations. Other base decision making on cardiac risk stratification and suggest different NIIT with or without stress-ECG according to level of risk. All of these guidelines seem not to suffice in reducing the low diagnostic yield of elective CA, as shown by Patel et al where only 37.6% of the elective CA without known CHD showed obstructive lesions [21].

### Strengths and limitations

Our study has several limitations. Our data originates from a single health insurance group in Switzerland, although the largest in the country. This is to our best knowledge the first European study on appropriateness of CA, especially in a non-gate-keeping health care system such as Switzerland. Most of the literature found on the matter originated from North America. Since this is a retrospective, observational study using administrative data, uncertainties exist concerning patient selection bias and unmeasured confounders affecting the likelihood of patients receiving NIIT. Since analyses originate in billing process, data not documented by the clinicians might be lacking. Also patients paying for care out of their own pocket due to a high deductible and not informing the insurance about performed diagnostic measures are not included in analysis. This proportion is likely to be neglectable, since CA always exceeds the deductible. Clinical data such as smoking status, cholesterol and blood pressure levels are not available. Lacking clinical data and therefore being able to estimate pre-test probability for CHD limits full estimation of appropriateness of diagnostic CA according to NICE and European Society of Cardiology guidelines, but not according to the American Heart Association. As a substitute for clinical data ATC and PCG are used, offering indirect information on comorbidities. Since the American Heart Association has recently discarded lipid target levels the sole information whether lipid lowering medication is prescribed seems to suffice as clinical information [22]. PCGs represent a strength since they have been shown to directly correlate with associated health care costs [16]. We chose a 2-Months window to assess the rate of NIIT prior to CA, possibly missing patients who underwent expanded medical therapy before sent to CA after medical therapy failed to improve symptoms. Nevertheless we consider our findings as robust due to following considerations: Since the recorded insurance claims cover almost all health care invoices, the data achieve a high degree of completeness. In order to verify the correctness of our findings we performed a sensitivity analysis by means of defining a subgroup of patients at high cardiac risk, which included patients having received therapeutic cardiac intervention within one month after diagnostic CA or within 18 months prior to CA prior. This analysis did not significantly change our main findings, indicated the unjustified large amount of patients without NIIT prior to elective CA.

### Implications for health service research and policy decision makers

The increasing use and associated health care costs [5] of CA in patients with stable CHD or non-cardiac chest pain together with the poor diagnostic yield of elective CA highlight the need to implement and unify and possibly improve available diagnostic algorithms to reduce inefficient and potentially ineffective care. The currently recommended algorithms based on clinical risk stratification do not suffice in reducing the low yield of CA in a non-emergency setting [18]. The physician consortium convened by the American Medical Association and the Joint Commission has identified elective percutaneous coronary intervention as one of the five unnecessary interventions which have to be aimed at reducing its use [23]. Patient selection should hence be contrived to such an extent that benefits of the procedure outweigh the risks, also taking associated costs into consideration. Considering that in our study population in a non-emergency setting every third patient undergoing elective CA with no therapeutic consequence had no NIIT before intervention suggests the need for more restrictive measures. A possible solution might be that invasive cardiologic centres should demand for NIIT prior to invasive diagnostic procedures. Less is sometimes more appropriate also in diagnostic CA, as seen in a study showing that patients in regions providing high-intensity care do not have better (and sometimes have worse) outcomes than those in regions providing low-intensity care [24].

## Conclusions

Our data show that despite the existence of guidelines a substantial overuse of a potentially harmful and inappropriate diagnostic intervention is performed suggesting the need for improvement of diagnostic pathways prior to invasive testing.
